# Outcomes of Patients with COVID-19 and Fungal Coinfections: A Systematic Review and Meta-Analysis Study

**DOI:** 10.30699/IJP.2024.2010087.3160

**Published:** 2024-02-15

**Authors:** Sadegh Khodavaisy, Haleh Sarrafnia, Alireza Abdollahi

**Affiliations:** 1 *Department of Medical Parasitology and Mycology, School of Public Health, Tehran University of Medical Sciences, Tehran, Iran*; 2 *Research center for antibiotic stewardship and antimicrobial resistance, Imam Khomeini Hospital Complex, Tehran University of Medical Sciences, Tehran, Iran*; 3 *Faculty of Biological Sciences, Islamic Azad University, Tehran-North Branch, Tehran, Iran*; 4 *Department of Pathology, School of Medicine, Imam Khomeini Hospital Complex, Tehran University of Medical Sciences, Tehran, Iran*

**Keywords:** COVID-19 associated pulmonary aspergillosis, COVID-19 associated candidiasis, Fungal coinfections

## Abstract

**Background & Objective:**

Fungal co-infections increase the incidence and mortality of viral respiratory tract infections. This study systematically reviews and conducts a meta-analysis to evaluate the prevalence of COVID-19 patients with fungal coinfections. The aim is to provide a concise overview of the impact of these infections on patient outcomes especially association with risk of mortality, informing future research and optimizing patient management strategies.

**Methods:**

To identify relevant studies on COVID-19 patients, we conducted a systematic search of databases from the beginning of the year until July 2023, including fungal co-infections, mortality, and sequelae. Eligibility criteria were developed using the PICO framework, and data extraction was carried out separately by two authors using standard techniques. Statistical analysis was performed using the correlation model and differences between studies were evaluated using the I2 test. R and RStudio were used for statistical analysis and visualization.

**Results:**

We initially identified 6,764 studies, and after checking for equivalence and consistency, 41 studies were included in the final analysis. The overall COVID-19 odds ratio for people who died from fungal infections was 2.65, indicating that patients infected with both COVID-19 and fungal infections had a higher risk of death compared to patients with COVID-19 alone. Specifically, COVID-19-associated pulmonary aspergillosis (CAPA) has a higher odds ratio of 3.36, while COVID-19-associated candidiasis (CAC) has an odds ratio of 1.84, and both are much more associated with death. However, coinfection of the fungus with other fungal species did not show a significant difference in the risk of mortality.

**Conclusion:**

This study identified CAPA and CAC as the most common infections acquired in healthcare settings. Fungal coinfections may be associated with an increased risk of death in COVID-19 patients.

## Introduction

COVID-19, which developed from the SARS-CoV-2 virus, triggered a global pandemic, forcing the World Health Organization to declare a public health emergency in early 2020. As of August 7, 2022, 581.8 million confirmed cases and 6.4 million deaths have been reported globally. According to a meta-analysis, around 17% of those infected with SARS-CoV-2 were asymptomatic, and those who were asymptomatic were 42% less likely to contract the virus. (1-4). However, uncertainties persist regarding reinfection rates and the duration of immunity. While some reports suggest varying rates of reinfection, the exact frequency remains uncertain. This raises concerns about the potential limitations of immunizations, as lifelong protection against the virus may not be guaranteed, and achieving herd immunity may be challenging if reinfections are prevalent (5-8). The prevalence of co-infections and super-infections in the global population remains uncertain. In the context of SARS-CoV-2, co-infections and super-infections typically involve community-acquired pathogens such as *Streptococcus pneumoniae, Haemophilus influenzae*, or* Staphylococcus aureus, *as well as hospital-acquired multidrug-resistant bacteria and fungi. These bacterial and fungal co-infections and super-infections can be significant complications of viral ailments and might result in worse outcomes (9-13). While co-infections are well-documented in influenza, SARS, MERS, and other respiratory viral diseases, evidence of bacterial and fungal interactions in SARS-CoV-2 remains limited and continues to evolve. Patients with COVID-19 face a significant risk of acquiring nosocomial co-infections during their hospital stay. Severe pneumonia is a common complication in SARS-CoV-2 patients, often requiring hospitalization, intubation, and transfer to the intensive care unit. Using broad-spectrum antibiotics to treat certain co-infections may raise the risk of acquiring multidrug resistance (9-16). According to reports, the proportion of microorganisms that are resistant to several medications is increasing globally. While respiratory failure due to SARS-CoV-2 infection is the primary cause of death, observations have demonstrated that hospitalized SARS-CoV-2 patients are also at heightened vulnerability to co-infections and super-infections. Importantly, both COVID-19-associated pulmonary aspergillosis (CAPA) and COVID-19-associated candidiasis (CAC) cases exhibited a considerably high mortality rate. Hence, we aimed to evaluate the impact of co-fungal infections on COVID-19 sufferers and their probability with chance of mortality.

## Material and Methods

The current study was executed based on the Preferred Reporting Items for Systematic Reviews and Meta-Analyses (*P*RISMA) guideline 2020 (17). 

**Search Strategy**

A systematic search was performed on electronic databases such as Web of Science, Scopus, and PubMed from January 2020 until July 2023. The search strategy included a combination of relevant medical subject heading (MeSH) terms and relevant keywords for (“COVID-19” OR “Sars-Cov-2” OR “Coronavirus”) AND (“Fungal infection” OR “COVID-19 associated pulmonary aspergillosis (CAPA)” OR Aspergillus*) AND (“COVID-19 associated candidiasis (CAC)” OR Candida*) AND (“Coinfections” OR “Healthcare settings”) AND (“Mortality” OR “Survival” OR “Death” OR “Outcome”). We also conducted a manual search on Google using the aforementioned keywords, selecting any more relevant studies.

**Eligibility Criteria**

This review study included both observational studies including cross-sectional studies, case-control studies, cohort studies, and clinical trials that investigated the outcomes of patients who also had fungal infections, notably CAC and CAPA. The study population will consist of patients diagnosed with COVID-19 who have been confirmed to have fungal coinfections, including CAC and CAPA. Patients may have acquired these infections in healthcare settings. In our study, fungal infections in the context of this review article will be defined as the presence of pulmonary aspergillosis and candidiasis. Other fungal infections, such as mucormycosis and pneumocystis, will not be considered within the scope of this study. We defined our eligibility criteria based on the PI(E)CO framework: (*P*) Population: COVID-19 patients. (E) Exposure: Fungal infection. (C) Comparison: Not applicable. (O) Outcome: Mortality. The primary outcome measures of interest will be mortality rates and any other relevant clinical outcomes associated with COVID-19 and fungal coinfections, including CAC and CAPA. Studies published in the English language and up to the present date will be considered for inclusion in the study. The exclusion criteria were defined as not having laboratory findings, not reporting mortality as an outcome, lack of individual data, and non-English language. 

**Data** **E** **xtraction and Outcome Measures**

Two unbiased authors accomplished the information extraction through the usage of a standardized sheet. Any dispute changed into resolved through a dialogue with a third party. The standardized sheet included: the authors’ names, year of publication, total number of participants, mean age of the participants, design of the study, and country of the study along with the number of deceased and alive in fungal infection groups and number of deceased and alive patients in those COVID-19 patients without fungal infection.

**Statistical Analysis and Data Synthesis**

The pooled odds ratios were calculated using the random effects model and Mantel-Haenszel method along with the 95% confidence intervals. For assessing the heterogeneity of the included studies, the I^2^ (I square) test was used. The Mantel-Haenszel method and random effects model were used for pooling the effect sizes. For testing the general importance of the random model, *z-*test was performed Potential publication bias was graphically assessed by creating funnel plots for each of the aforementioned groups. RStudio (RStudio, Inc., Boston, MA) and R (R Foundation for Statistical Computing, Vienna, Austria) were used for the statistical analysis and creation of forest and funnel plots.

## Results

**Figure 1:**
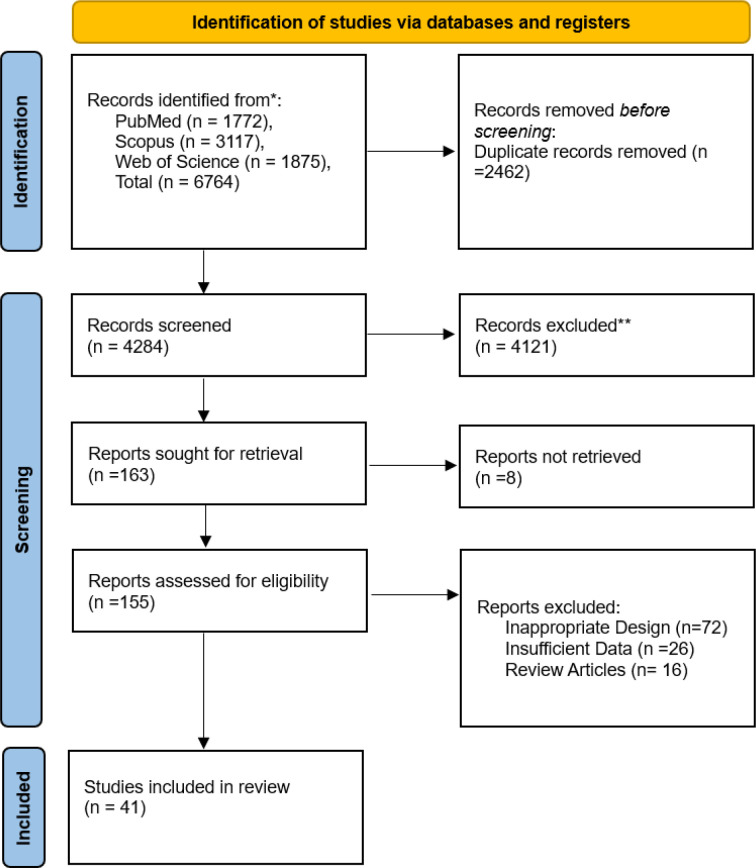
PRISMA flow diagram of the systematic search and study selection

**Figure 2:**
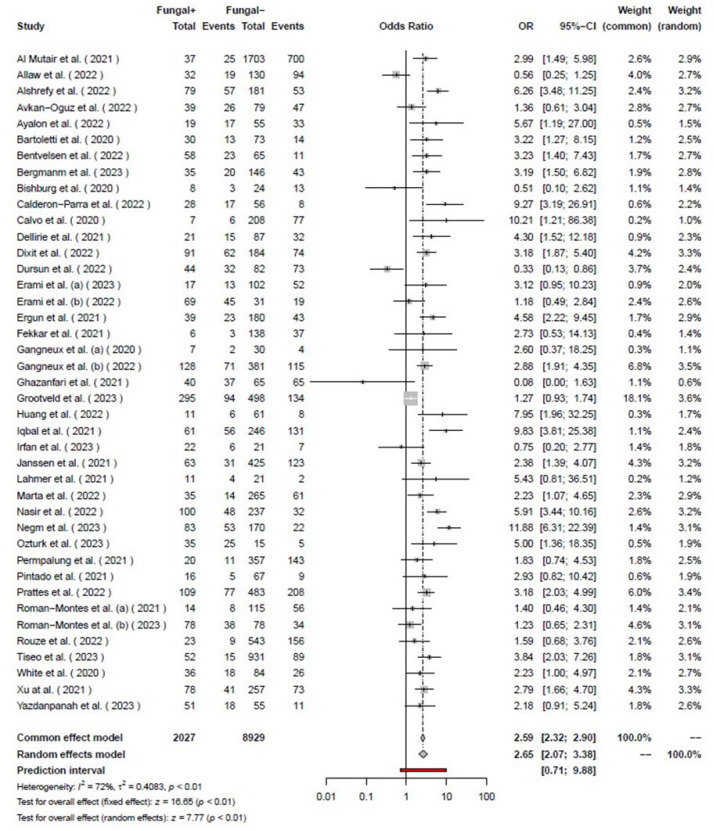
Forest plot the risk of mortality of the studies among COVID-19 patients with and without fungal infections.

**Figure 3:**
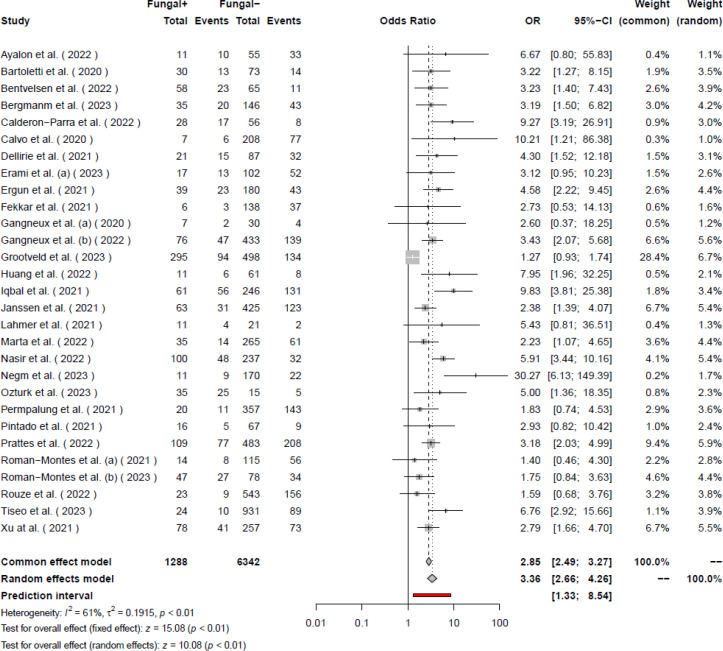
Forest plot of the studies with COVID-19 patients with *Aspergillus* spp. infection.

**Figure 4:**
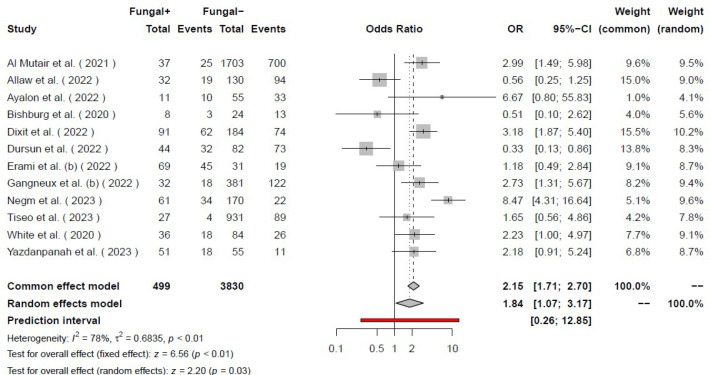
Forest plot of the studies with COVID-19 patients with *Candida* spp. infection.

**Figure 5:**
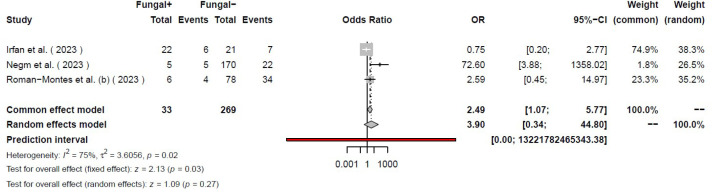
Forest plot of the studies with COVID-19 patients with other fungal infections.

**Figure 6:**
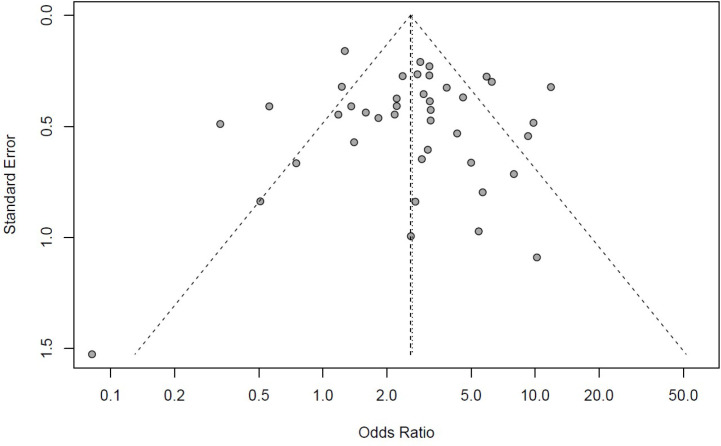
The funnel plot represents a potential bias across the studies.

Our systematic search of the literature included 6,764 studies, primarily. After removing the duplicates (2,462), 4,284 studies were analyzed. Finally, 155 studies were included for full-text evaluation. Based on our inclusion and exclusion criteria, 41 studies (18-58) were included in our final meta-analysis model (Figure 1). The pooled odds ratio of COVID-19-associated mortality with fungal infection was 2.65 (2.07 – 3.38). Based on the random effects model the odds of mortality were significantly higher (*P*<0.01) among those with COVID-19 and fungal coinfection compared to those who were only infected with COVID-19. Further details regarding each specific study and the common effects model are available in Figure 2. The pooled odds ratio of mortality with CAPA was 3.36 (2.66–4.26). Based on the random effects model the odds of mortality were significantly higher (*P*<0.01) among those with COVID-19 and *Aspergillus *species coinfection compared to those who were only infected with COVID-19 (Figure 3). The pooled odds ratio of mortality with CAC was 1.84 (1.07 – 3.17). Based on the random effects model the odds of mortality were significantly higher (*P=*0.03) among those with COVID-19 and *Candida *species coinfection compared to those who were only infected with COVID-19 (Figure 4). The pooled odds ratio of COVID-19 associated mortality with fungal infection with other fungi was 2.65 (2.07–3.38) which mortality did not show any significant difference (*P= *0.27) among those with COVID-19 and fungal coinfection compared to those who were only infected with COVID-19 (Figure 5). Also, further information regarding the publication bias is summarised in the funnel plot of the included studies (Figure 6).

## Discussion

The most reported fungal infections were CAPA and CAC, while other fungal infections were exceptionally rare. CAPA, caused by *Aspergillus* species, and CAC, caused by *Candida* species, were primarily acquired in healthcare settings and were more frequently observed in prospective studies. Retrospective studies reported even higher mortality rates, possibly due to challenges in promptly recognizing these infections. These infections were primarily acquired in healthcare settings and were more frequently documented in prospective observations. Retrospective studies reported even higher mortality rates, possibly due to challenges in promptly recognizing these infections. Our findings showed a substantial association between fungal coinfections and higher mortality in COVID-19 patients. The pooled odds ratio for COVID-19-associated mortality with fungal infection was 2.65, indicating a higher risk of death compared to those infected with COVID-19 alone. CAPA had a higher odds ratio of mortality at 3.36, while CAC had a lower odds ratio of 1.84. Other fungal infections did not show a significant difference in mortality compared to COVID-19 alone. However, it is crucial to emphasize that the majority of studies focused primarily on CAPA, limiting the overall evidence on the impact of fungal infections on mortality. Nonetheless, CAC also had a notable impact on mortality. Nevertheless, CAC also contributed significantly to mortality, albeit with a lower odds ratio (OR) (59-62). This review represents valuable insights into the prevalence and characteristics of CAC and CAPA in severe COVID-19. The prevalence of CAC varied based on the study population and geographical region, with *Candida albicans* and *Candida auris* being the most prevalent organisms. The mortality rate of invasive *Candida* infections varies according to the species and antifungals used for therapy. Similarly, the prevalence of CAPA varied among ICU patients with COVID-19, with *Aspergillus fumigatus* being the most prevalent fungus (63, 64). The case-fatality rate for CAPA was substantial. The majority of studies focused on CAPA, limiting the overall evidence on the impact of fungal infections. Nonetheless, CAC also had a notable impact on mortality (18, 23, 26). This comprehensive review represents the first extensive examination of fungal infections in severe COVID-19, providing valuable insights into the prevalence and characteristics of CAC and CAPA, which are essential for understanding mortality rates and specific Candida species involved (65-68). Lansbury* et al.* identified four fungal pathogens from three studies in COVID-19 patients. *C. albicans* was isolated from the respiratory tract in multiple patients and from the urinary tract in one patient. Additionally, *Aspergillus flavus* and *A. fumigatus* were associated with CAPA, while *C. glabrata* caused CAC. However, their analysis primarily concentrated on bacterial coinfections, revealing that COVID-19 cases with any coinfection had an advanced liability of mortality compared to controls. In another study by Musuuza* et al.,* the frequency of fungal coinfections was reported at 4, and superinfections were set up in 8 of the COVID-19 cases across 35 studies. Both coinfections and super-infections were more current in ICU cases. Aspergillus spp. was the most constantly reported microorganism among coinfections, while Candida spp. was the primary cause of fungal superinfections. The study revealed that cases with any viral, bacterial, or fungal infection had3-fold advanced odds of dying, with no significant differences grounded on etiology. still, detailed characteristics and causative sub-analyses of cases with fungal infections weren't handed (69- 71). Also, Peng* et al.* Investigated fungal coinfections in COVID-19 cases from the ICU. Their analysis included critically ill cases and those with mixed populations. The proportion of fungal coinfections was reported, and the mortality rate associated with fungal coinfections and COVID-19 was determined (25, 51, 55). The overall mortality rate was found to be 10.9%. Several studies have explored the presence and impact of fungal coinfections in COVID-19 patients, primarily focusing on pathogens like *Aspergillus* spp., and *Candida* spp. Patients with any coinfection or superinfection had a higher risk of mortality. However, further research is needed to understand the specific characteristics and outcomes related to fungal infections in the context of COVID-19 (59, 72-75). The timely identification of fungal infections is of utmost importance to initiate appropriate and prolonged treatment. It is crucial to distinguish infections from colonization and employ reliable diagnostic methods to minimize the risk of misdiagnosis and inappropriate therapy. Diagnosing fungal infections in the clinical setting presents challenges such as work overload, understaffing, and organizational issues arising from the pandemic. These factors can impede accurate microbiological testing and specimen collection, increasing the likelihood of diagnostic errors. Prompt and accurate diagnosis of fungal infections is crucial for initiating appropriate and prolonged treatment. Furthermore, severely unwell sufferers infected with each SARS-CoV-2 and different pathogens face a poor prognosis, regardless of the unique etiology of the infections. Hence, screening for CAPA or CAC needs to be taken into consideration for almost all severely unwell patients, no matter their capability susceptibility to fungal infections (25, 27, 32, 47, 56). Various diagnostic methods, such as ELISA- or PCR-based detection of fungi in bronchoalveolar lavage samples, can accelerate the diagnosis of fungal infections. Serum galactomannan testing can also be used but may have limitations in sensitivity. Culture remains the gold standard but may miss some infections. Standardized approaches and unified terminology are essential for accurate and timely detection of fungal infections in COVID-19 patients. Given the complex challenges associated with diagnosing fungal infections in COVID-19 patients, a coordinated and standardized approach is essential to ensure accurate and timely detection, ultimately improving patient outcomes (24, 28, 38, 45).

## Conclusion

Our findings suggest a higher mortality rate in COVID-19 patients with fungal coinfections. These findings highlight the necessity of incorporating CAPA and CAC within COVID-19 management plans.
